# A Review of Canadian Diagnosed ADHD Prevalence and Incidence Estimates Published in the Past Decade

**DOI:** 10.3390/brainsci12081051

**Published:** 2022-08-08

**Authors:** Stacey D. Espinet, Gemma Graziosi, Maggie E. Toplak, Jacqueline Hesson, Priyanka Minhas

**Affiliations:** 1 CADDRA—Canadian ADHD Resource Alliance, Toronto, ON M5A 3X9, Canada; 2 Department of Psychology, York University, Toronto, ON M3J 1P3, Canada; 3 Faculty of Education, Memorial University of Newfoundland, St. John’s, NL A1C 5S7, Canada; 4 Department of Psychology, University of Calgary, Calgary, AB T2N 1N4, Canada

**Keywords:** ADHD, prevalence, epidemiology, prevalence, incidence, Canada

## Abstract

(1) Background: ADHD is recognized as one of the most common neurodevelopmental disorders. The worldwide prevalence of ADHD is estimated at 5.3%; however, estimates vary as a function of a number of factors, including diagnostic methods, age, sex and geographical location. A review of studies is needed to clarify the epidemiology of ADHD in Canada. (2) Methods: A search strategy was created in PubMed and adapted for MEDLINE and PsycINFO. Papers were included if they examined diagnosed ADHD prevalence and/or incidence rates in any region of Canada, age group and gender. A snowball technique was used to identify additional papers from reference lists, and experts in the field were consulted. (3) Results: Ten papers included in this review reported on prevalence, and one reported on incidence. One study provided an overall prevalence estimate across provinces for adults of 2.9%, and one study provided an overall estimate across five provinces for children and youth of 8.6%. Across age groups (1 to 24 years), incidence estimates ranged from 0.4% to 1.2%, depending on province. Estimates varied by age, gender, province, region and time. (4) Conclusions: The overall Canadian ADHD prevalence estimate is similar to worldwide estimates for adults. Most studies reported on prevalence rather than incidence. Differences in estimates across provinces may reflect the varying number of practitioners available to diagnose and prescribe medication for ADHD across provinces. To achieve a more comprehensive understanding of the epidemiology of ADHD in Canada, a study is needed that includes all provinces and territories, and that considers estimates in relation to age, gender, ethnicity, geographical region, socioeconomic status and access to mental healthcare coverage. Incidence rates need further examination to be determined.

## 1. Introduction

Attention deficit hyperactivity disorder (ADHD) is recognized as the most common neurodevelopmental disorder of childhood. A frequently cited study by Polanszky of 2007 reported a worldwide ADHD prevalence estimate of 5.3% [1]. Studies carried out in countries other than Canada showed a range of ADHD estimates. For instance, estimates for children include 7.5% in Australia [2] and 10.2% in the United States [3]. Two studies on adolescents in China and Africa provided estimates of 6.3% [4] and 7.5% [5], respectively. One German study on children and adolescents combined provides an estimate of 6.1% [6]. Estimates are typically lower for adults than for children and adolescents [7]. A 2021 survey conducted in the United States, for example, found an ADHD prevalence rate for adults of 4.25% [8]. Findings from two recent worldwide meta-analyses suggest a prevalence of 3.4% in children and adolescents [9], and a lower prevalence in adults of 2.6% [10].

In addition to potential differences between countries, prevalence estimates may also vary due to the methods used and sample characteristics. Additionally, while prevalence estimates indicate the number of existing cases of a disorder in the population (existing + new cases over total population), incidence estimates indicate the number of new cases being added (i.e., new cases over total (susceptible) population). When the duration of a disorder is short, and new instances are constant or increasing, incidence can trend higher than prevalence. However, when the duration of a disorder tends to be lifelong (as is the case for ADHD), prevalence can trend higher than incidence because new cases are added and existing cases remain. Thus, prevalence and incidence provide unique information which combined, help to clarify the epidemiology of ADHD.

A few studies have examined the prevalence and/or incidence of ADHD specifically in the Canadian population, but these findings have not yet been summarized. To clarify the epidemiology of ADHD in Canada, a review of existing studies on the prevalence and incidence of diagnosed ADHD is needed. The aim of this review is to summarize Canadian ADHD prevalence and incidence estimates published in the past ten years and to consider these in relation to demographic (i.e., age and gender), geographic and methodological factors.

## 2. Materials and Methods

A search strategy to identify relevant literature using Boolean operators was developed using PubMed and adapted for Medline, and PsycInfo with the following terms: (ADHD or ADD or AD/HD or Attention *) and (Canada or Canadian or province * or territory or territory *) and (epidemiology, prevalence, incidence). All studies were screened, and data were abstracted by two reviewers. A snowball technique (i.e., examining reference lists of selected papers) and experts were consulted to identify additional relevant papers. Papers published within the past 10 years (2012 to 2022) were included if they provided estimates of diagnosed ADHD prevalence or incidence based on data from the Canadian population, regardless of age group, gender or geographical location examined. Papers that focused on symptoms of ADHD without a diagnosis (e.g., self-reported attention difficulties) and that focused on a particular environmental condition (e.g., ADHD with greenspace exposure or traumatic brain injury) were excluded.

## 3. Results

### 3.1. Description of Included Papers

A total of ten papers are included in the review [11,12,13,14,15,16,17,18,19,20], all of which were published from 2012 to 2022 (see Table 1). Ten papers included prevalence estimates of ADHD in Canada [11,12,13,14,15,16,17,18,19,20]. Out of these papers, one also examined the incidence of ADHD in Canada [15]. One additional review paper [21] that was initially included, was ultimately excluded because it did not provide prevalence or incidence estimates. This paper was reviewed to ensure that any relevant studies mentioned were included here. See Appendix A for a PRISMA diagram [22] outlining results of the search strategy used to retrieve articles for the current paper.

This group of studies based estimates on data collected from 1999 [11,20] to 2015/6 [12,17,20]. The range for data collection was similar for papers that focused on ADHD prevalence in children and/or adolescents (1999–2015/6) [11,15,17,18,20] and those that examined prevalence in adults (1999–2018) [13,14,15,16,19]. With regard to data collection methods used to derive estimates, some papers used primary care EMR data [12] and health administrative databases [15,16,17,18,20], whereas others used national/provincial surveys [11,13,14,19]. When papers included multiple prevalence or incidence estimates, the most recent estimate was used for this review [11,12,15,16,17,20].

The papers with prevalence estimates included children alone [11,17], adults alone [13,14,16,19], and a combination of children and adults [12,15,18,20]. All six of the papers that examined ADHD prevalence in children included preschoolers (i.e., children under 6 years old) and school-aged children (between 6 to 12 years old), and five included adolescents (i.e., 13 to 17 years old) [12,15,17,18,20]. Four papers focused on adults between the ages of 18 to 65 years old [12,13,14,19]. The one paper that provided incidence estimates included a combination of children, adolescents and adults (1999–2012) [15].

Four of the papers provided overall prevalence estimates across Canadian provinces using national datasets [12,13,14] or nationally administered surveys [11]; four provided prevalence estimates for a single province [16,17,18,20]; and one provided prevalence and incidence estimates for four provinces individually (Nova Scotia, Manitoba, Quebec and Ontario) [15]. One did not specify geographical location [19].

Regarding gender, while all papers acknowledged gender differences in ADHD prevalence, six provided separate estimates for males and females [11,12,14,15,17,18]. The paper that included incidence estimates differentiated between males and females, although no exact estimates were provided [15]. No other non-binary gender identity was noted in any of the papers. The ethnic background of the participants was reported in only one paper; the majority were classified as “white” [13].

The diagnosis of ADHD was established based on different informants and methods. Some relied on physician reports [15,16,17,18]; others relied on self-reporting [13,14,19,20] or parent/caregiver-report [11]. Only one study [12] used an algorithm to identify ADHD based on information from the participants’ primary care electronic medical records. Six of the studies used data collected from health administrative datasets [12,15,16,17,18,20], and the remaining used survey data [11,13,14,19]. Not all of the papers specified which criteria were used to determine an ADHD diagnosis [11,13,14]. Five of the studies used ICD-9 diagnostic criteria [12,15,16,17,20], and five used ICD-10 criteria [15,16,17,18,20]. One paper referred to DSM-IV criteria [19].

### 3.2. ADHD Prevalence

#### 3.2.1. Overall ADHD Prevalence in Canada

None of the included studies provided an overall national prevalence estimate collapsed across age groups (i.e., children, adolescents and adults combined). However, one study provided an overall estimate of 2.9% across a large age range (i.e., 20 to 64 years of age), gender (i.e., male/female) and all provinces [14]. 

#### 3.2.2. Age Differences

The one study that provided an estimate for children alone (aged 3 to 9 years) indicated that ADHD prevalence across provinces is approximately 1.1% for preschoolers and 4.1% for school-aged children [11]. Of the studies that provided ADHD prevalence estimates for children and adolescents combined [12,15,17,18,20], estimates ranged from 2.6% [17] to 8.6% [12]. Only one of these studies provided an overall estimate across provinces (i.e., Alberta, Manitoba, Newfoundland, Ontario and Quebec) of 8.6% [12]. The studies that examined ADHD prevalence amongst adults in Canada [12,13,14,15,16,19] provided estimates ranging from 2.7% or 2.9% (across all provinces) [13,14] to 7.3% (across five provinces) [12]. One study provided separate estimates of ADHD prevalence for young adults (18 to 34 years, 7.3%) and older adults (35 to 64 years, 5.5%) [12]. Among a specific population of relatively old (i.e., majority of the sample aged 50 and over) Canadian Forces members and veterans, ADHD prevalence was estimated at 3.3% (geographical location not specified) [19].

A few of the studies indicate that compared to adults and preschoolers, ADHD diagnosis is higher in school-aged children [11,12,15,17,20] and young adults [12]. In particular, one study found that the highest overall annual prevalence rates are in children 5 to 14 years of age, which held true across time (1999–2012) and province (Manitoba, Ontario, Quebec, Nova Scotia) [15].

#### 3.2.3. Gender and Ethnicity Differences

The subset of papers including children indicates that the prevalence of ADHD is approximately twice as high amongst males compared to females [11,12,15,16,17,18]. Estimates for males range from 3.7% [11] to 13.3% [20]. Estimates for females range from 1.5% [11] to 7.0% [12]. Three studies suggest that this gender gap persists into adulthood [12,16,18], with estimates for adult males ranging from 5.8% [12] to 10.3% [16], and for adult females ranging from 2.6% [16] to 6.5% [12].

None of the studies looked at differences in prevalence by ethnic background. One study compared differences between first and second-generation immigrants in British Columbia with a non-immigrant sample [20]. Prevalence was highest for the non-immigrant sample (9.2%), followed by the second-generation sample (5.9%). The lowest prevalence rate was found for the first-generation sample (4.3%).

#### 3.2.4. Socioeconomic and Regional Differences

Only one study looked at Canadian ADHD prevalence rates in relation to socioeconomic status (SES) and region (urban versus rural) [16]. This study did not find an SES gradient in ADHD diagnosis, but when restricted by region, a small negative gradient was found for urban living and a small positive gradient for rural living. Additionally, adults in the highest income bracket were less likely than those in other income brackets to receive a diagnosis of ADHD before age 18.

#### 3.2.5. Provincial Differences

One study looked at differences in estimates across provinces and indicated that ADHD prevalence may be higher in certain provinces compared to others [15]. Findings were that ADHD prevalence amongst children is higher in Nova Scotia (3.8%) and Quebec (3.8%) compared to Manitoba (2.8%) and Ontario (1.1%). Amongst adults, prevalence is also higher in Nova Scotia (1.7%) compared to Manitoba (0.8%), Quebec (0.7%) and Ontario (0.5%).

#### 3.2.6. Differences across Time

A number of the studies reviewed here indicate that ADHD diagnosis and the number of patients prescribed ADHD medication are increasing over time [11,12,15,17]. One study reported that the prevalence of ADHD diagnosis in Canada has increased amongst all age groups—4 to 17-year-olds (6.9–8.6%), 18 to 34-year-olds (5.7–7.3%), and 35 to 64-year-olds (5.2–5.5%)—from 2008 to 2015, particularly for children and young adults [12]. Another study indicated that this upward trend in diagnosis holds across provinces. The highest increase in ADHD prevalence across time (1999 and 2012) was found for Quebec (3.5%) [15]. This study suggests that this increase was associated with a shift across time in practitioner type primarily diagnosing ADHD, from specialists to general practitioners [15].

### 3.3. ADHD Incidence

#### 3.3.1. Overall ADHD Incidence in Canada

None of the papers included in this review provided an incidence rate for ADHD across all age groups or using a national sample.

#### 3.3.2. Age Differences

The one paper that examined ADHD incidence did so across a wide age range (1 to 24 years) [15]. Findings were that incident ADHD diagnosis is highest in 5 to 9-year-olds (from 0.8% in Ontario to 2.1% in Quebec) and 10 to 14-year-olds (0.5% in Ontario to 1.5% in Quebec) compared to all other age groups examined. This held true across year of study (1999 to 2012) and province examined (Manitoba, Nova Scotia, Ontario, Quebec).

#### 3.3.3. Provincial Differences

Across age groups, the highest incidence estimate was found for Quebec (1.2%), followed by Nova Scotia (1.0%), Manitoba (0.8%) and Ontario (0.4%) [15].

#### 3.3.4. Differences across Time

Based on one study, there is some indication that ADHD incidence is rising across time (1999 to 2012) in Manitoba, Nova Scotia and Quebec but remaining stable in Ontario [15].

See Table 1 for articles regarding the prevalence and incidence estimates of ADHD in Canada.

### 3.4. Which Practitioners Are Diagnosing and Treating ADHD in Canada

**Figure 1:**
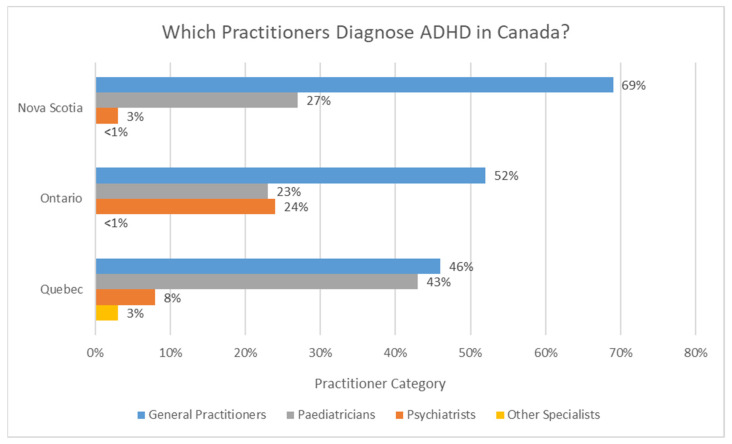
ADHD diagnosis by practitioner type in the following Canadian provinces (2011): Nova Scotia, Ontario and Quebec. Adapted from [15].

According to Vasiliadis et al. (2017) [15], who reported on the incidence of ADHD in Canada, ADHD diagnoses are primarily made by general practitioners in the three provinces examined (Nova Scotia, Ontario and Quebec). In Nova Scotia, diagnoses seem to be primarily performed by general practitioners (69%), followed by pediatricians (27%) and then psychiatrists (3%) [15]. In Ontario, diagnoses also seem to be primarily performed by general practitioners (52%), then psychiatrists (24%), pediatricians (23%), and other specialists (<1%) [15]. In Quebec, the primary sources of incident ADHD diagnoses seem to be general practitioners (46%) and pediatricians (43%), followed by psychiatrists (8%) and other specialists (3%) [15]. These statistics are displayed in Figure 1.

**Figure 2:**
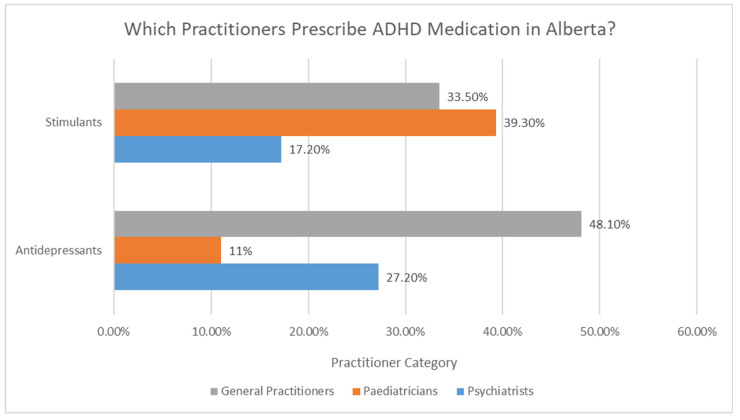
Prescription patterns for ADHD by practitioner type in Alberta (2015). Adapted from [17].

Only one study in this review [17] examined medication treatment trends for ADHD by practitioner type (in Alberta), as shown in Figure 2. Findings from this study indicate that pediatricians most frequently prescribe stimulants (39.3%), followed by general practitioners (33.5%).

Psychiatrists prescribe stimulants least frequently (17.2%). This is in contrast to prescriptions for antidepressants, of which most are provided by general practitioners (48.1%), followed by psychiatrists (27.2%) and then pediatricians (11%).

Another study included in this review looked at medications prescribed to patients with ADHD aged 1 to 24 years, and found that the majority (70%) are prescribed stimulant medication [18]. The study indicated that psychiatric consultation is positively associated with antidepressant and antipsychotic medication prescriptions. The presence of a comorbidity (i.e., having a diagnosis of anxiety or depression) and increased age were also found to be positively associated with antidepressant prescriptions.

## 4. Discussion

The aim of this review was to examine the prevalence and incidence rates of diagnosed ADHD in Canada in the past decade. Ten papers were included in this review that covered all provinces and age groups from preschoolers to older adults. Based on one paper across five provinces, overall ADHD prevalence in children and youth (ages 4 to 17 years) was estimated to be 8.6% [12], and based on one paper across all provinces, overall ADHD prevalence in adults (ages 20 to 64 years) is estimated to be 2.9% [14], which is similar to worldwide estimates in adults [10]. Findings from this review indicate that:

- The prevalence of diagnosed ADHD varies across provinces (from 0.5% in Ontario to 3.8% in Nova Scotia).
- The prevalence of ADHD and prescription of ADHD medication have increased over time (1999–2015) for all age groups and provinces (particularly Quebec with an increase in prevalence of 3.5% in just over a decade) [11,12,15,17].
- Prevalence is higher amongst children and adolescents than adults, both within individual provinces and across Canada [12,15,18].
- Prevalence is higher amongst males than females at all ages, although the disparity may decrease with age [11,17,18,20].
- The incidence of diagnosed ADHD in children and youth varies across provinces (from 0.4% in Ontario to 1.2% in Quebec) [15] and has increased over time.

ADHD prevalence and incidence estimates require further examination, as only two studies in the past decade have examined prevalence across all provinces. No study covers all provinces and territories, nor all age groups. These are important gaps, as findings from the group of studies reviewed here highlight significant variability in estimates by province and age, e.g., [9]. All studies included here reported information on prevalence, but only one reported on incidence. Further estimates are needed, particularly of incidence, to clarify the number of individuals affected and the rate of growth of individuals with ADHD in the population. For a comprehensive understanding of the epidemiology of ADHD in Canada, a study is needed that includes data from all provinces and territories, and that looks across all age groups, taking a lifespan approach.

There are a number of future directions and questions that should arise from this review. While some of the studies looked at estimates in relation to gender, only the dichotomy of males versus females was used. Additionally, only one study included here reported on ethnicity as a variable. Importantly, one study in this review highlighted variability in prevalence rates related to immigrant and refugee status [20], and another emphasized the need to consider socioeconomic and regional (urban vs. rural) differences [16]. Another important factor needing attention in epidemiological research is the influence of mental health insurance coverage on access to service access [16]. Further research on these demographic and socioeconomic variables will help clarify the diverse needs of individuals with ADHD across the country and inform service delivery. Consideration of comorbid conditions will also be important for future studies, as approximately 65–80% of children and 85% of adults with ADHD have at least one comorbid psychiatric disorder (anxiety, depression, conduct disorder, etc.). Individuals with ADHD have a 13-year shorter estimated life expectancy due to both psychiatric and medical comorbidity [23].

Canadian studies on prevalence in the past decade indicate an upward trend in ADHD diagnosis and medication treatment across time and provinces. One possible explanation is that variations in study methodology play a role, as found in a recent systematic review and meta-regression analysis of worldwide studies [21]. It was concluded that variations in estimates across those studies can be largely explained by methodological differences, rather than time or geographical location. Methodological variability is notable both within and across the studies included in this review, particularly with regard to data sources and case determination. A unique contribution of this review is that all studies examined diagnosed ADHD. Screening studies that asked about symptoms related to ADHD without a diagnosis by a health professional were excluded because the presence of self-reported symptoms alone does not confirm a diagnosis of ADHD and can result in inflated estimates [24]. However, a few of the studies included here relied on self- or other-reported diagnosis. Only six studies out of ten based the presence of ADHD on a diagnosis performed by a health professional and recorded in the health system [12,15,16,17,18,20]. To achieve a more accurate reflection of the prevalence/incidence of ADHD in Canada, it is important that future studies include only confirmed and documented cases of ADHD.

Differences in taxonomy used to identify ADHD across studies and within studies, across time, may also influence estimates. Some research suggests that prevalence estimates based on the International Classification of Diseases (ICD) may underestimate ADHD prevalence compared to those that rely on Diagnostic and Statistical Manual of Mental Disorders (DSM) criteria [24,25,26,27,28]. Additionally, diagnostic criteria change across time, potentially influencing estimates, such as the change in symptom onset and exclusion criteria from DSM-IV to DSM-V. Important to note is that this review captured only the prevalence of individuals diagnosed with or treated for ADHD, rather than the prevalence of all diagnosed and undiagnosed individuals with ADHD. For instance, individuals diagnosed in the school and mental health systems would not be captured in the six studies included here that relied on health administrative datasets [12,15,16,17,18,20]. As discussed by Vassiliadis et al., prevalence estimates based on physician claims may reflect a suspected or working diagnosis rather than a confirmed one. Administrative datasets may also miss ADHD diagnoses because comorbidities of anxiety and depression are often present with ADHD, and claims may be made under billing codes for these conditions instead of ADHD [15]. While the use of a wide range of health administrative datasets and publicly accessible surveys across the country highlights opportunities for gathering comprehensive epidemiological data on ADHD nationwide, it may be ideal to link data from multiple sources to most accurately inform case identification.

Provincial differences in ADHD prevalence/incidence and treatments (e.g., prescriptions of stimulants and antidepressants) raise several questions regarding the types of services and number of practitioners available to diagnose and treat ADHD. While provincial differences may be an important factor influencing prevalence, Canada also has an additional consideration given its geography, including large distances between communities and the resulting impact on access to services [29]. However, the one study in this review that looked at regional differences found that those in rural or lower-income communities are more likely to be diagnosed with ADHD and medicated [16]. This finding may depend, in part, on the extent to which ADHD is viewed as a medical condition across regions and provinces [11], as well the extent to which parents prefer accessing ADHD support through medical settings versus school and community settings [11].

Upward trends across time in ADHD diagnosis and treatment may reflect improvements in diagnosis and in ADHD medications (e.g., the introduction of long-acting stimulants in 2003), since the use of stimulants is considered first-line treatment for ADHD. This trend may also be associated with the increased availability of mental health training for primary care practitioners and practice guidelines supporting the successful management of ADHD in primary care [30,31]. Consistent with this possibility, findings from one study reviewed here found a shift across time and provinces away from ADHD diagnosis, primarily from specialists to general practitioners [15]. This shift also coincides with practitioner-reported improvements in comfort and willingness to diagnose and treat ADHD in primary care [11,12,17,18,31]. While combined medication and psychosocial treatment is recognized as an important treatment approach for ADHD, the insufficient availability of specialty care resources and publicly-funded psychosocial treatments in Canada limits treatment options, which may point to an important gap in equitable ADHD care access in Canada [11]. More data are needed on diagnosis and treatment trends over time, across Canadian provinces, territories and regions, to inform future efforts regarding training practitioners and ensuring access to services [29,31].

## 5. Conclusions

The prevalence of ADHD in Canada based on studies published in the past 10 years varies depending on age group, gender, location and methodological approach.

Future Canadian ADHD prevalence and incidence research would benefit from:

- Overall estimates across provinces and territories.
- Estimates moderated by age, gender, ethnicity, geographical location (e.g., province/territory and urban vs. rural), socioeconomic status and access to mental health care insurance.
- Standardized methods for defining ADHD cases, particularly with regard to diagnostic criteria (e.g., DSM and ICD) and data sources (e.g., record of a diagnosis by a health professional)
- ADHD case identification using a validated algorithm based on pre-established criteria (currently considered best practice for case determination when using large primary care datasets) [12].
- Incidence estimates tracked with demographic data.

## Figures and Tables

**Table 1:** Summary of ten research articles related to the prevalence and incidence estimates of ADHD in Canada.

Name of Study	Age Range	Geographic Location	Sample	Statistic	Prevalence Estimate	Incidence Estimate	Case Definition	Data Source	Gender	Ethnicity
Braut & Lacourse (2012) [11]	Children: 3 to 9 years	All provinces	Three cross-sectio nal samples of nonreferred children, 1994–1995: *n* = 12,595, 2000–2001: *n* = 13, 904, and 2006–2007: *n* = 14,655. (Number of individuals with ADHD not reported.)	Number of children with ADHD over total number of children, both overall and for each subgroup.	Overall prevalence from 2000 to 2007: 1.7% to 2.6%. Preschoolers: 0.5% to 1.1%, School-age children: 2.2% to 4.1%.	—	The presence of a child’s psychiatric diagnosis of ADHD was reported by the parent most knowledgeable about the child.	National Longitudin al Survey on Children and Youth. Household s were selected through the Statistics Canada’s Labour Force Survey	Male: 51.0% Female: 49%	—
Morkem, Han delman, Queenan, Birtwhistl, & Barber. (2020) [12]	Children and youth: 4 to 17 years, Young adults: 18 to 34 years, Adults: 35 to 64 years	Alberta, Manitoba, Ontario, Quebec, Newfoundland	Any patient, 2008 to 2015, who received care from their primary care clinic in the year of study or the preceding year, *N* = 19,683, *n* = 246 with ADHD.	The case definition of ADHD was applied to each yearly practice population *n* to produce a count of those with ADHD; number of patients with ADHD over total number of patients for each given year.	Prevalence from 2008 to 2015 4- to 17- year olds: 6.9% to 8.6%, 18- to 34-year olds: 5.7% to 7.3%, 35- to 64-year-olds: 5.2% to 5.5%.	—	Patient was 4 years of age or older and either (a) the medical record included ICD-9 code 314 in one or more visits, and one or more prescriptions of ADHD-related medications; or (b) the medical record included ICD-9 code 314 in two or more visits. The EMR algorithm was validated by conducting a manual electronic chart review of a sample of 492 patients by a blinded abstractor.	Canadian Primary Care Sentinel Surveillance Network (CPCSSN) (a repository of primary care EMR data)	Male: ~42.9% Female: ~57.1% (Gender distribution was not reported in the paper)	—
Connolly, Speed, & Hesson. (2016) [13]	Adults: 20 to 64 years	All provinces	Population-based sample, 2012, *N* = 17,311, *n* = 377 with ADHD	Total number of respondents who said that they have been diagnosed with ADD/ADH D over total number of respondents.	Overall prevalence: 2.7%.	*–*	As part of the CCHS-MH interview, respondents were asked, “Do you have attention deficit disorder?”	Public Use Microdata File of the 2012 Canadian Community Health Survey (CCHS; Statistics Canada, 2013)	Male: 52.1% Female: 47.9%	White: 80.6% Non-White: 19.4%
Hesson & Fowler (2018) [14]	Adults: 20 to 64 years	All provinces	Population-based sample, 2012, *N* = 16,957, *n* = 488 with ADHD	Total number of respondents who said that they have been diagnosed with ADD/ADHD over total number of respondents.	Overall prevalence: 2.9%.	–	As part of the CCHS-MH interview, respondents were asked, “Do you have attention deficit disorder?”	Public Use Microdata File of the Canadian Community Health Survey–Mental Health (CCHS-MH ) 2012 (Statistics Canada, 2013)	Male: 58.8% Female: 41.2%	—
Vasiliadis, Diallo, Rochette, Smith, Langille, Lin, et al. (2017) [15]	Children and Youth: 1 to 17 years, Young adults: 18 to 24 years	Manitoba, Ontario, Quebec, Nova Scotia	Young adults who received a Primary diagnosis of a mental disorder between 1999 and 2012. (*N*, total sample size, and *n*, number of individuals with ADHD, not reported.)	Incidence and prevalence were calculated yearly. Annual prevalence: proportion of persons who had received a primary diagnosis of ADHD in a given year. Annual incidence: proportion of new cases in the year who had not previously received an ADHD diagnosis.	Annual age-standardized prevalence from 1999 to 2012 1- to 17-year-olds: Nova Scotia: 2.2% to 3.8%, Manitoba: 1.5% to 2.8%, Quebec: 1.1% to 3.8%, Ontario: 1.1% and 1.1%. 18-to 24-year-olds Nova Scotia: 0.5% to 1.7%, Manitoba: 0.2% to 0.8%, Quebec: 0.1% to 0.7%, Ontario: 0.2% and 0.5%.	Incidence from 1999 to 2012 1- to 24-year-olds: Nova Scotia: 0.8% to 1.0%, Manitoba: 0.6% to 0.8%, Quebec: 0.5% to 1.2%, Ontario: 0.5% to 0.4%.	At least 1 physician visit or hospitalization within a given year with the following primary diagnoses: 314 for ICD-9 or the equivalent ICD-10 code (F90.x). Diagnoses could be performed by general practitioners, paediatricians, psychiatrists, or other specialists.	Administra tive linked patient data from Manitoba, Ontario, Quebec, and Nova Scotia. Obtained from the same sources as the Canadian Chronic Diseases Surveillance Systems (Med-Ech o in Quebec, the Canadian Institute of Health Information Discharge Abstract Database in the 3 other provinces, plus the Ontario Mental Health Reporting System).	—	—
Yallop, Brownell, Chateau, Walker, Warren, Bailis et al. (2015) [16]	Adults: 18 to 29 years	Manitoba	Cross sectional analysis of adults, 2007/08 to 2008/09, *N* = 207,544, *n* = 14,762 with ADHD.	Number of people with ADHD diagnosis over total study population.	Overall lifetime prevalence: 7.1%.	*–*	Lifetime prevalence of Diagnosis determined from physician visits and hospitalizations, using the ICD-9-CM of 314 (hyperkinetic syndrome of childhood) or the ICD-10-CA code of F90 (hyperkinetic disorders). In addition, people who had 2 or more prescriptions for a psychostimulant and no diagnosis for conduct disorder, narcolepsy, or catalepsy.	The Manitoba Population Health Research Data Repository	Male: 50.5% Female: 49.5%	*–*
Leung, Kellett, Youngson, Hathaway & Santana (2019) [17]	Children and youth: 18 years of age or under	Alberta	Population-based sample, 2015, *N* = 144,243. (*n*, number of individuals with ADHD, not reported.)	Prevalence was calculated yearly. Annual prevalence: number of cases in cohort each year over annual provincial population, multiplied by 1000 to obtain rates per 1000 people.	Prevalence from 2008 to 2015 Females: 3.1% to 3.9% Males: 8.0% to 9.5%.	*–*	Child (age ≤ 18 years) with at least one physician visit or hospitalization with a primary diagnostic code corresponding to one of the psychiatric disorders of interest (ICD-9 or ICD-10).	Retrospective analysis of six administrati ve databases, 2008–2015, Alberta: Discharge Abstract Database (DAD), Practitioner Claims Database, National Ambulatory Care Reporting System (NACRS, since 2010), Alberta Ambulatory Care Reporting System (AACRS, before 2010), Provincial Registry, and Pharmaceutical Information Network (PIN).	Male: ~ 59.3% Female: ~ 40.7%	—
Hauck, Lau, Wing, Kurdyak & Tu (2017) [18]	Children and youth: 1 to 24 years	Ontario	Population-based sample, 2002–2012, *N* = 10,000, *n* = 536 Individuals with ADHD.	Number of definite cases of ADHD in the cohort over total number of included cases.	Overall prevalence: 5.4% Males: 7.9% Females: 2.7%.	—	Charts in which the family physician recorded a diagnosis of ADHD (reason for visit ICD10 diagnosis was F00 to F99 OR X60-X84), if a neuropsychological test or report indicated a diagnosis, or if correspondence from a school/school board indicated a diagnosis of ADHD.	Medical records contained in the Medical Record Administrati ve data Linked Database (EMRALD).	Male: 50.6% Female: 49.4%	—
Sareen J, Bolton SL, Mota N, et al.: (2018) [19]	60% over 50 years old (no other data reported on age of sample)	No data on geographic location of sample provided	*N* = 2941 Canadian Forces members and veterans, 2018	Total number of participants who have been previously diagnosed with ADHD over the total number of participants.	Overall prevalence 2018: 3.3%	—	Self-report health professional diagnosis of ADHD based on DSM-IV	Two-wave 2002–2018 Canadian Armed Forces Members and Veterans Mental Health Follow-up Survey (CAFVMHS)	Male: 87.8%; Female: 12.2%	—
Gadderm an, Petteni, Janus, Puyat, Guhn & Georgiades (2022) [20]	Children and youth: Birth to 19 years	British Columbia	Population- based sample, 1996–2016, *N* = 470,464 Non-immigrant, comparison sample: *n* = 307,902 (*n*, non-immigrant sample with ADHD, not reported, *n* = ~ 53,914) Refugee: *n* = 19,686 Immigrant: *n* = 142,011	Total number of participants who have been previously diagnosed with ADHD over the total number of participants. Overall estimates adjusted for years living in British Colombia.	Overall prevalence from 1996 to 2016: Non-immigrant, comparison sample: 3–5 years: 1.3% 6–12 years: 9.2% 13–19 years: 7.0% First-generation immigrant: 3–5 years: 0.8% 6–12 years: 4.3% 13–19 years: 2.1% Second-generation immigrant: 3–5 years: 0.9% 6–12 years: 5.9% 13–19 years: 3.7% Refugee (first-generation): 3–5 years: 0.6% 6–12 years: 4.1% 13–19 years: 2.4% Refugee (second-generation): 3–5 years: 1.1% 6–12 years: 6.2% 13–19 years: 3.7%	–	To identify indicators of ADHD diagnoses, implemented adapted criteria used by the Manitoba Centre for Health Policy, which includes a combination of ICD-9-CM and ICD-10 codes from the hospital discharge records and practitioner billing records.	Health administrative records from 1996 to 2016, BC PharmaNet, and Immigration, Refugees, and Citizenship Canada’s (IRCC) Permanent Resident Database.	Male: 51.7% Female: 48.3%	–

**Figure A1:**
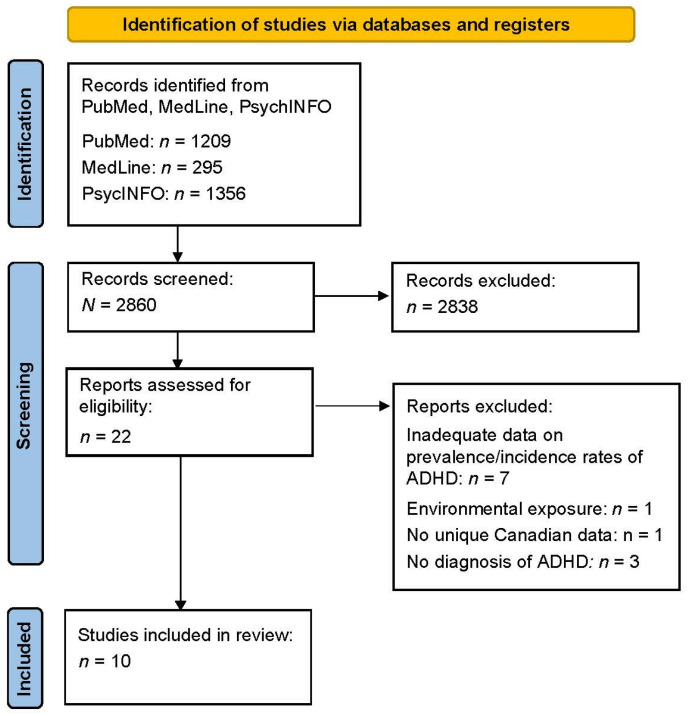
PRISMA diagram of the search strategy that was used to retrieve articles for this paper. Adapted from [22].

## Data Availability

Not applicable.
